# Unexplored hypersaline habitats are sources of novel actinomycetes

**DOI:** 10.3389/fmicb.2014.00242

**Published:** 2014-05-27

**Authors:** Polpass Arul Jose, Solomon Robinson David Jebakumar

**Affiliations:** Department of Molecular Microbiology, School of Biotechnology, Madurai Kamaraj UniversityMadurai, India

**Keywords:** actinomycetes, hypersaline environments, antibiotic, *Streptomyces*, drug discovery

Pathogenic microorganisms have evolved sophisticated mechanisms to inactivate antibiotics and rendered an urgent need for new antibiotics that would target the emerging multidrug-resistance (Butler et al., 2013). Consequently, search for novel sources of potent antibiotics is desperately needed to develop potent drugs. Microbial resources have made an incredible contribution to the antibiotic drug discovery and development process over the last seven decades (Demain and Sanchez, 2009). In particular, actinomycetes are the most important source of bioactive natural compounds with a long track record of producing novel molecules, including several commercially important drugs like Streptomycin, Gentamicin, Vancomycin, Clindamycin, Erythromycin, Amphotericin, Rifampicin, and Tetracycline. More novel molecules with potential therapeutic applications are still on the row to be discovered from these actinomycetes (Baltz, 2007).

Microbial assemblages and their species distributions in specific environments are mostly determined by their specific environmental conditions, which may translate into novel chemistry (Genilloud et al., 2011). The actinomycetes that produce antibiotics are abundant in soil. In recent decades, isolation and exploitation of actinomycetes for novel compounds from conventional environments have led to rediscovery of known compounds (Walsh, 2003; Tulp and Bohlin, 2005). However, diverse actinomycetes from poorly studied unusual environments promises a raise in the prospect of discovering novel compounds with potential activities that can be developed as a resource for drug discovery (Peraud et al., 2009; Jose et al., 2011; Poulsen et al., 2011; Carr et al., 2012; Subramani and Aalbersberg, 2012; Hamedi et al., 2013; Jose et al., 2013; Yuan et al., 2014). In this respect, current actinomycetes isolation programs are reoriented toward largely unexplored, unusual and extreme environments like hypersaline marine environments, extreme inland saline zones, volcanic zones, hyperarids and glaciers (Hamedi et al., 2013; Jose and Jebakumar, 2013b).

The extreme habitats are characterized by chemical or physical conditions that differ significantly from those found in environments that support more abundant and varied life forms (Zhao et al., 2011). Hypersaline environments are typical extreme habitats, in which the high salt concentration, alkalinity, and low oxygen concentrations are environmental factors that may limit their biodiversity (Ventosa, 2006). There are several reports on inhabitance of microorganisms including actinomycetes in diverse hypersaline environments such as salt lakes (Swan et al., 2010; Guan et al., 2011; Phillips et al., 2012), solar salterns (Baati et al., 2008; Sabet et al., 2009), salt mines (Chen et al., 2007) and brine wells (Xiang et al., 2008). However, these hypersaline environments remain largely unexplored as a source of novel actinomycetes in many tropical and subtropical regions throughout the world. In a recent review, Hamedi et al. (2013) described that actinomycetes form a stable, metabolically active and persistent population in various marine hypersaline ecosystem and it is accepted that halophilic actinomycetes will provide a valuable resource for novel products of industrial interest, including antimicrobial, cytotoxic, neurotoxic, antimitotic, antiviral and antineoplastic activities. Series of bioactive metabolites like Pyrostatins, Salinosporamides, Abyssomicins, Trioxacarcin A, Gutingi- mycin, Sporolides, Marinomycins, Himal- omycins, Diazepinomicin, Helquinoline, Lajollamycin, Tetrodotoxin, Mecherchar- mycins, Cyanosporasides, Erythronolides, and Ammosamide D have been reported from this physiological group of actinomycetes (Hamedi et al., 2013). In continued efforts to discover novel natural products from new extremophilic actinomycetes, Zhao et al. (2011) discovered new linear polyketides, actinopolysporins A, B, and C, as well as the known antitneoplastic antibiotic tubercidin from the halophilic actinomycete *Actinopolyspora erythraea* YIM 90600. Tian et al. (2013) reported the isolation and characterization of *p*-Terphenyls with antifungal, antibacterial, and antioxidant activities from halophilic actinomycete *Nocardiopsis gilva* YIM 90087. Jang et al. (2013) declared a significant discovery of new antibiotic, anthracimycin, which is produced by a marine-derived actinomycete in saline culture. More recently, an antimicrobial quinoline alkaloid has been isolated from a novel halophilic actinomycete, *Nocardiopsis terrae* YIM 90022 (Tian et al., 2014).

In accordance with the above reports, we initiated a research program which was designed to discover new natural products from novel actinomycetes originating from previously unexplored hypersaline environments. Saltpan soil samples were collected aseptically from saltpans of coastal solar saltern ponds at Arumuganeri, Tuticorin (about Latitude 8.43 N and Longitude 78.60 E) and inland solar salterns at Sambhar Lake, Jaipur (about Latitude 26.94561 N and Longitude 75.20968 E) in India. Physicochemical characteristics from this study confirmed that this environment is a hypersaline zone. In the course of our program, diverse actinomycetes were isolated by using selective isolation methods which employed stamping, heat and dilution, and succeeded with isolation of both *Streptomyces* (common actinomycete) and non-*Streptomyces* including rare actinomycetes *Nonomuraea, Actinoalloteichus* and *Pseudonocardia* (Jose and Jebakumar, 2012, 2013a). A total of 83 actinomycetes were isolated and assigned to eight genera (*Streptomyces, Micromonospora, Nocardia, Nocardiopsis, Nonomuraea, Saccharopolyspora, Pseudonocardia* and *Actinoalloteichus*) on the basis of their 16S rDNA sequences and it was the first step toward better understanding of actinomycete community from solar saltern ponds in India. Our preliminary biological activity screening and subsequent structural characterisation experiments suggested the occurrence of antimicrobial compounds producing *Streptomyces* and rare actinomycetes and confirmed that hypersaline solar salterns are reservoirs of antibiotic producing actinomycetes (Jose and Jebakumar, 2013a, b). The range of antimicrobial activities exerted by different actinomycete isolates were summarized in Table 1. In concise, our continuing study contributes in acquaintance of solar saltern associated actinobacteria and further augments the array of actinomycetes available for antibiotic discovery programs.

**Table 1:** **Range of antimicrobial activities of the actinomycete isolates obtained from Indian inland (Jose and Jebakumar, 2013a) and coastal (Jose and Jebakumar, 2013b) solar salterns**.

**Isolates**	**Antimicrobial activities**							
	**Antibacterial activity**					**Antifungal activity**		
	**Gram—positive**		**Gram—negative**			***Aspergillus niger***	***Fusarium oxysporum***	***Alternaria alternata***
	***Staphylococcus aureus***	***Bacillus subtilis***	***Klebsiella pneumoniae***	***Proteus vulgaris***	***Salmonella typhi***			
*Streptomyces* sp. JAJ06	+	+	+	+	+	−	−	−
*Streptomyces* sp. JAJ07	+	+	+	+	+	−	−	−
*Streptomyces* sp. JAJ13	+	+	+	+	+	−	−	−
*Nonomuraea* sp. JAJ18	+	+	+	+	+	−	−	−
*Streptomyces* sp. JAJ19	+	+	+	+	+	−	−	−
*Micromonospora* sp. JAJ20	+	+	+	+	+	+	+	+
*Streptomyces* sp. JAJ28	−	−	−	−	−	+	+	+
*Streptomyces* sp. JAJ41	+	+	−	−	−	−	−	−
*Streptomyces* sp. JAJ59	+	+	+	+	+	−	−	−
*Actinoalloteichus* sp. JAJ70	+	+	+	+	+	+	+	+
*Streptomyces* sp. JAJ73	−	−	−	−	−	+	+	+
*Pseudonocardia* sp. JAJ77	+	+	+	+	+	−	−	−
*Pseudonocardia* sp. JAJ82	−	−	−	−	−	+	+	+

+, positive for antimicrobial activity; −, negative for antimicrobial activity.

-In conclusion, escalating reports on discovery of diverse natural compounds from halophilic and halotolerant actinomycetes which inhabit in hypersaline environments suggested that this physiological group has enormous capacity to produce array of secondary metabolites with disparate activities. Indeed, hypersaline environments warrant significant attention as habitats of novel actinomycetes and unlock new avenues for natural products discovery.

## Conflict of interest statement

The authors declare that the research was conducted in the absence of any commercial or financial relationships that could be construed as a potential conflict of interest.
